# Sustainable Surveillance of Neglected Tropical Diseases for the Post-Elimination Era

**DOI:** 10.1093/cid/ciab211

**Published:** 2021-06-14

**Authors:** Hollie-Ann Hatherell, Hope Simpson, Rebecca F Baggaley, T Déirdre Hollingsworth, Rachel L Pullan

**Affiliations:** 1 Department of Disease Control, London School of Hygiene and Tropical Medicine, London, United Kingdom; 2 Department of Respiratory Sciences, University of Leicester, Leicester, United Kingdom; 3 Big Data Institute, University of Oxford, Oxford, United Kingdom

**Keywords:** neglected tropical diseases, Africa, post-elimination, surveillance, survey

## Abstract

The World Health Organization’s (WHO’s) 2030 road map for neglected tropical diseases (NTDs) emphasizes the importance of strengthened, institutionalized “post-elimination” surveillance. The required shift from disease-siloed, campaign-based programming to routine, integrated surveillance and response activities presents epidemiological, logistical, and financial challenges, yet practical guidance on implementation is lacking. Nationally representative survey programs, such as demographic and health surveys (DHS), may offer a platform for the integration of NTD surveillance within national health systems and health information systems. Here, we describe characteristics of DHS and other surveys conducted within the WHO Africa region in terms of frequency, target populations, and sample types and discuss applicability for post-validation and post-elimination surveillance. Maximizing utility depends not only on the availability of improved diagnostics but also on better understanding of the spatial and temporal dynamics of transmission at low prevalence. To this end, we outline priorities for obtaining additional data to better characterize optimal post-elimination surveillance platforms.

Ensuring effective post-intervention and elimination surveillance is a challenge that faces all neglected tropical diseases (NTDs) programs [1, 2]. As successful preventive chemotherapy (PC) and intensified disease management (IDM) interventions drive down infection prevalence and/or disease incidence, remaining affected individuals become more unevenly distributed across space and populations. Outlier areas and population groups become more prominent for transmission and potentially harder to find, and larger samples over broader areas are required to confirm trends [2]. Consequently, determining the optimal design of stand-alone and integrated surveillance approaches both during program implementation and prior to verification of elimination has been an urgent priority [3]. Once programs reach elimination targets, however, there is little consensus or guidance on how best to maintain effective surveillance [4]. This operational gap risks leaving countries vulnerable to undetected recrudescence, which could lead to resurgence of infections and undo years of progress in NTD control.

Due to the pressing need to provide clear guidance to programs that are reaching elimination targets, we now need greater focus on post-elimination surveillance: how can we identify recrudescence in a timely way and at a reasonable cost? What scale of sampling, at what frequency, and among which sentinel populations are required? Here, we discuss these issues and outline key requirements for post-elimination surveillance platforms and their relevance across the range of NTDs currently flagged by the World Health Organization (WHO) for elimination, either in terms of transmission or as a public health problem, by 2030. This review is restricted to the WHO Africa region because this is the region with the highest burden of NTDs and represents a case study that illustrates the questions that need to be answered for all countries with active NTD programs approaching or anticipating elimination.

## What Does Elimination Mean in the Context of NTDs?

Individual targets for NTD eradication and elimination as a public health problem have been hugely influential in driving progress; committing governments, donors, and health workers to focus on these neglected diseases; and facilitating unprecedented levels of support from pharmaceutical companies. As part of its road map for reducing the burden of NTDs, the WHO has identified eradication or elimination targets for 13 diseases, although for most this does not mean reducing prevalence or incidence to zero [1]. Only 2 diseases (yaws and dracunculiasis) are targeted for eradication, and an additional 3 (human African trypanosomiasis gambiense, leprosy, and onchocerciasis) for interruption of transmission. The remaining 8 are targeted for elimination as a public health problem, the definition of which varies substantially between diseases, from achieving zero or very low case fatality (rabies and visceral leishmaniasis) to reducing prevalence of moderate and heavy infection (soil transmitted helminthiasis [STH] and schistosomiasis). For additional details of these goals, see Supplementary Table 1.

In most instances, NTD programs, be they for PC or IDM diseases, have not been integrated within routine activities performed by primary care structures but instead run as parallel interventions, often with the support of a range of partners and stakeholders. To ensure sustainability and contribute to health system strengthening, there are now increasing calls to replace these vertical structures with routine, integrated surveillance of NTDs in endemic countries [5–7]. Opportunities for integration and mainstreaming and the ongoing relative importance of passive and active surveillance systems, will depend not only on stated elimination goals but also on key disease characteristics, particularly the pathogen life cycle and transmission route, the proportion of infections that result in severe disease, the relevance of asymptomatic cases for transmission, the availability of diagnostic tools for asymptomatic infections, and the public health response that would be triggered by the detection of an infectious case.

Broadly, for NTDs controlled through individual case detection and management (IDM NTDs, including leprosy and rabies), incredibly low population prevalence post-elimination make stand-alone surveys unworkable. Instead, post-validation surveillance will remain dependent on reports triggered by symptomatic case presentation, with the public health response centered on treatment of the individual case along with other investigations, such as contact tracing, source identification, and/or community sensitization [1, 8–10]. This becomes increasingly challenging as countries achieve elimination targets, as has been demonstrated for leprosy and yaws, both of which have had long-standing elimination and eradication goals. In many instances, these diseases are no longer seen as a public health priority, resource allocations have been reduced and expertise lost, cases have become increasingly clustered among marginalized populations with low accessibility, and progress has stagnated [1, 2].

A recent history of renewed investment and strengthened public–private partnerships [3], combined with calls for enhanced integration [4], have regalvanized IDM programs globally. Task-shifting of case identification and management closer to affected communities, coupled with training of and motivation for healthcare workers, strengthened reporting, better diagnostics and treatments, and innovative active case finding strategies, are all likely to play pivotal roles if elimination goals are to be successfully achieved. Crucially, however, once reached, these efforts (and the investments required to achieve them) will need to be broadly sustained to prevent reemergence.

For PC-NTDs, opportunities for post-elimination surveillance may vary depending on the disease in question. For STH and schistosomiasis, achievement of elimination as a public health problem does not mean the end of PC interventions; as a result, resources and infrastructure for stand-alone surveillance (eg, through school-based surveys) will potentially be safeguarded for the post-elimination era. In contrast, PC interventions for lymphatic filariasis, onchocerciasis, and trachoma stop after verification of elimination; as a result, surveillance to detect resurgence is more likely to require systems external to existing NTD programs. For these diseases, transmission is driven by cases of asymptomatic and subclinical infections [5, 6]; consequently, in post-elimination settings, an active surveillance system to identify these infections would be required. The detection of an infectious case would trigger a public health response that might include mass treatment of the community or wider area in which the case was identified [4], but specific management of the individual infected would not be necessary. The primary health system may detect some additional infections [7], but its main role would be in providing management and rehabilitation services to patients affected by the sequelae of past infections and in monitoring the numbers of these patients (which are also indicators for the verification of elimination of diseases such as lymphatic filariasis and trachoma [11]).

## Toward Practical Integrated Surveillance

In many NTD-endemic countries, passive surveillance systems already face issues of underresourcing, low representativeness due to uneven levels of access and availability of health services, and low sensitivity of clinical diagnosis, which will worsen as clinical expertise is lost due to reduced disease incidence. To ensure effective post-elimination surveillance of NTDs, it will be essential that the NTD community remains focused on strengthening the primary healthcare system and improving access to care. However, large-scale, active surveillance platforms will additionally be required for the surveillance of PC-NTDs.

Nationally representative survey programs such as demographic and health surveys (DHS) may offer an appropriate platform for active surveillance, and already play an important role in epidemiological monitoring of infectious diseases including malaria and human immunodeficiency virus (HIV) [12]. These programs are typically conducted by in-country institutions with external technical and financial assistance and are characterized by enhanced data infrastructure and well-organized testing capabilities. To assess the feasibility and utility of leveraging nationally representative survey programs for NTD post-elimination surveillance, we reviewed key characteristics of the major population-based survey programs in Africa.

Surveys were identified using information from the Global Health Data Exchange [13]. We considered all representative health-monitoring survey programs currently taking blood samples ( “blood surveys”), chosen because these already have an infrastructure available for the collection and analysis of biomarkers, and all DHS regardless of sample collection (as the largest institution conducting nationally-representative surveys), with an assumption that biomarker collection may be an additional module that could be integrated into future surveys.

We identified 185 blood surveys conducted between 1997 and 2019 (surveys ongoing at the time of analysis were excluded) in the WHO African region. These included DHS, AIDS, and malaria indicator surveys (AIS and MIS) and special surveys (all conducted through the DHS program by USAID [14]); multiple indicator cluster surveys (MICS, conducted by UNICEF [15]); population-based HIV impact assessments (conducted by ICAP [16]); and STEPwise chronic disease risk factor surveillance surveys (conducted by WHO [17]). The included surveys represented 45 out of 47 countries in the WHO African region. There were no surveys recorded for Guinea-Bissau or South Sudan.

For the second dataset, we included 227 DHS conducted between 1986 and 2020. Survey types included AIS, DHS, MIS, MICS, and special surveys and included 42 countries in the WHO African region. There were no surveys recorded for Algeria, Guinea-Bissau, Mauritius, Seychelles, or South Sudan.

### Survey Design and Frequency

Impact assessment surveys for monitoring the status of PC-NTD control programs prior to achieving elimination are usually designed as community- or school-based cluster surveys representative at the level of the implementation unit (typically an administrative or health district) or an evaluation unit representing a group of implementation units with similar transmission characteristics [18–20]. Rather than measure prevalence to a given level of precision, they are usually intended to classify units as above or below a target prevalence threshold. For diseases including onchocerciasis and schistosomiasis, purposive sampling is recommended in areas of expected high transmission [8], which is intended to increase the likelihood of identifying foci of infection, but at the cost of survey representativeness.

In contrast, DHS-style surveys are generally designed to be representative at the national level, the residence level (urban–rural), and the regional level (departments, states), with enumeration areas selected systematically within strata with probability proportional to size. The indicators they measure require smaller sample sizes for representativeness at these levels than do surveys for NTDs [9], suggesting that data collected through this platform may be too sparse to detect foci of recrudescence of public health importance. However, they may be able to provide a platform for surveillance if certain adaptations to increase precision can be integrated.

Adaptations to conventional survey design might include additional adaptive or snowball sampling and spatial oversampling in areas predicted to be at higher risk of transmission using geostatistical models [10]. Adaptive sampling may include testing of household or community members of identified cases [21], which is appropriate for NTD surveillance since many of these diseases show strong spatial clustering. Model-based geostatistical (MBG) analysis frameworks allow for incorporation of spatial correlation and environmental covariates and can lead to significant predictive gains. Notably, MBG approaches are increasingly applied to improve inference from malaria data collected through MIS platforms [22, 23]. Additional data on the extent of spatial heterogeneity observed at different spatial scales in very low transmission settings for NTDs are essential if we are to fully evaluate this utility further.

A second consideration is the periodicity of sampling. Although the optimal survey frequency for post-elimination NTD surveillance (balancing timely detection of recrudescence with surveys costs) remains to be determined and is an important research priority, recommended post-MDA surveillance for STH currently uses 3-year surveys and that for LF involves 2- to 3-year surveys in the immediate post-validation era. We therefore assumed that programs that conducted at least 3 surveys in the past 10 years would provide the minimum frequency required to detect recrudescence of NTDs at a level of public health importance and examined the frequency of DHS against this threshold.

**Figure 1.**
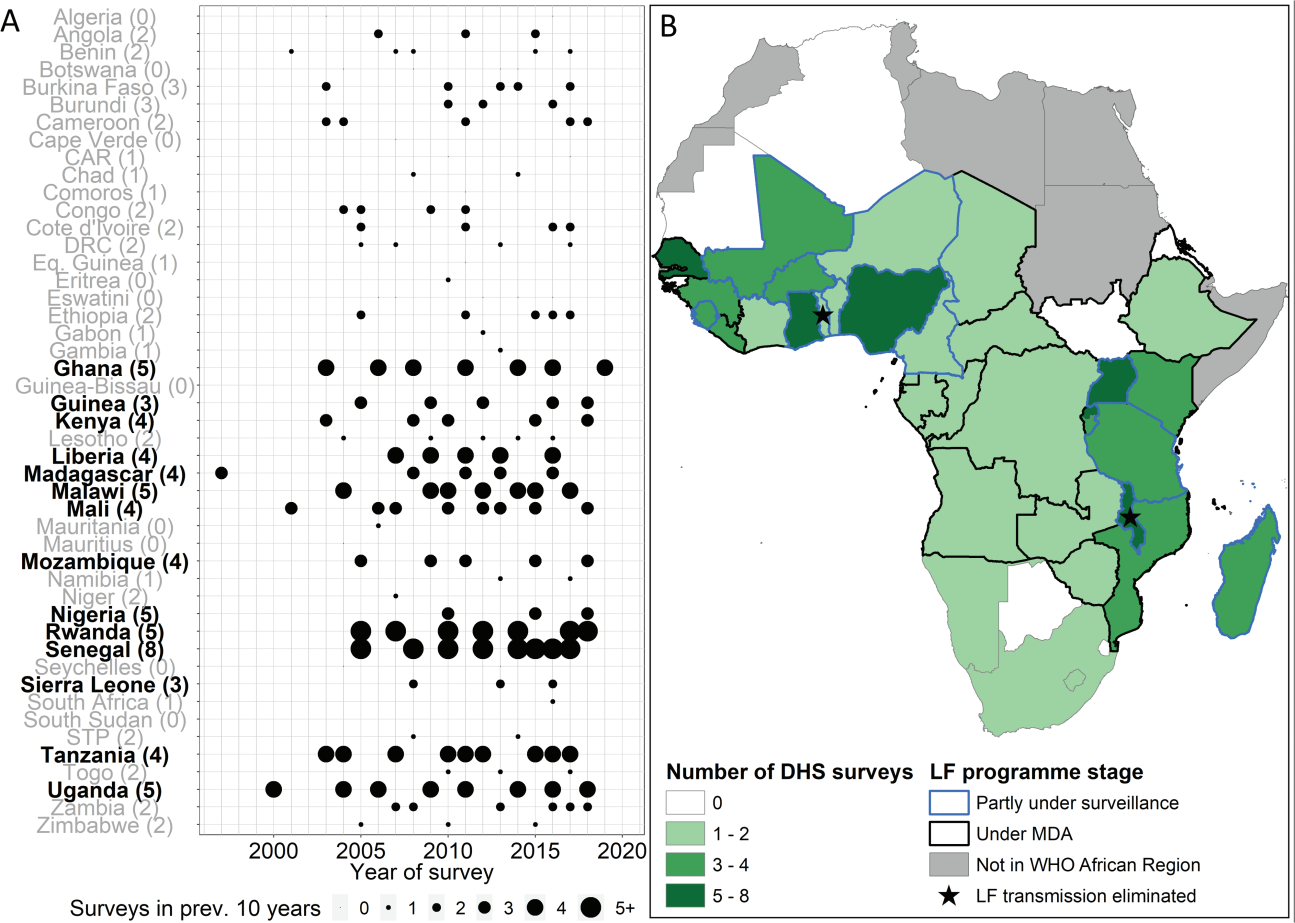
Frequency of DHS by country in the WHO Africa region. Dots indicate years in which a DHS was conducted. The size of each dot reflects the number of years out of the previous 10 in which a DHS took place. The number of surveys conducted in 2008–2018 is shown in brackets. Countries with more than 3 surveys in 2008–2018 (ie, surveys at least roughly every 3 years) are highlighted in bold. African countries are colored according to the number of DHS surveys conducted in 2008–2018. Survey frequency is compared with status of LF control in each country, as defined by surveillance and MDA status using data from the Expanded Special Project for Elimination of Neglected Tropical Diseases. Abbreviations: CAR, Central African Republic; DHS, demographic and health survey; DRC, Democratic Republic of Congo; LF, lymphatic filariasis; MDA, mass drug administration; STP, Sao Tome and Principe; WHO, World Health Organization.

Figure 1 provides a summary of DHS conducted by country, with the number of surveys performed in each country in the previous 10 years provided as a measure of survey frequency. Twenty-five countries undertook 3 or more DHS in the past 10 years, and 14 had only 1 or no surveys over this time. The mapped data in Figure 1 illustrate the large area of central Africa in which countries currently under mass drug administration (MDA) or partly under post-MDA surveillance for lymphatic filariasis, used as an illustrative example, have survey rounds of insufficient frequency for adequate post-elimination surveillance. This demonstrates that there cannot be sole reliance on DHS for effective surveillance without a substantial increase in survey frequency in all countries at risk of recrudescence. However, additional research, including multiyear cross-sectional studies in post-elimination settings, and mathematical modeling will be required to determine the minimum frequency of post-elimination surveillance to detect recrudescence at levels of public health importance for different NTDs in the longer term.

### Age Ranges and Sentinel Populations

Appropriate target populations for post-elimination NTD surveillance depend mainly on the age groups or populations most at risk, although these may vary across different transmission settings [24]. An additional consideration is that detection of antibodies for an infection in any age group born since verification of elimination indicates post-elimination transmission and would be a key criterion for public health action.

For schistosomiasis and STH, target product profiles (TPPs) of diagnostics for post-elimination surveillance have recommended targeting children aged 6–14 years [25], while those for trachoma surveillance recommended focusing on children aged 1–5 years, although evidence from modeling studies has suggested a target population of children aged 1–9 years, which is in line with guidelines for monitoring of programmatic implementation [26]. For lymphatic filariasis, post-elimination surveys have generally targeted wider age ranges, although children aged <2 years have rarely been sampled in the past, and sometimes activities have been focused on children [4]. Mathematical modeling of onchocerciasis transmission indicates that the most appropriate target age group depends on age-specific exposure patterns in different contexts: surveillance of children aged 0–9 years is recommended in areas where exposure increases rapidly from birth, while surveillance of children aged 5–14 years is recommended where exposure increases at a slower rate [24].

**Figure 2.**
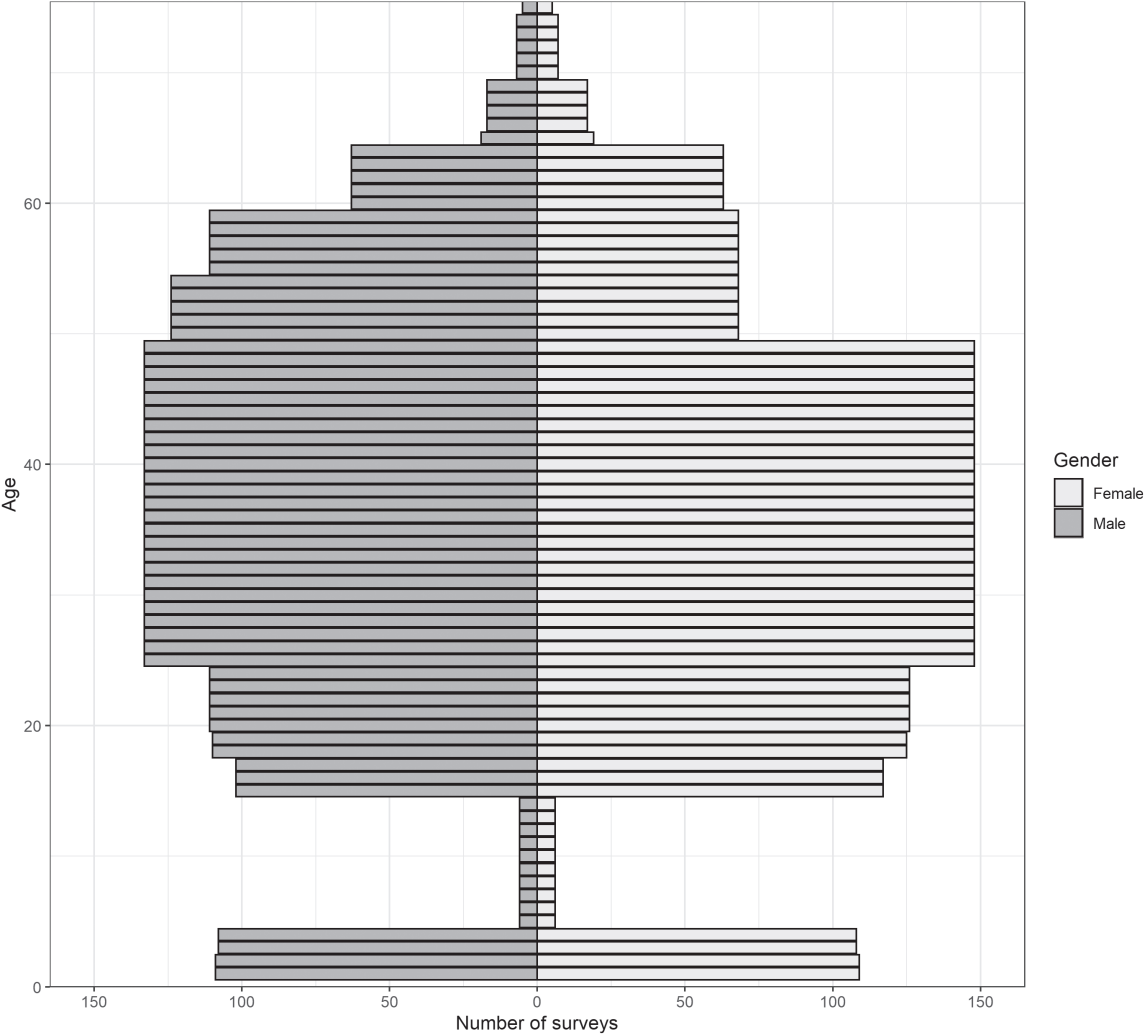
Representation of males and females by age in surveys that collected blood samples conducted in the African region.

Figure 2 shows the representation of males and females by age in surveys that collect blood samples conducted in the WHO African region, illustrating the underrepresentation of school-age children in all nationally representative surveys in the region. This is because they principally test for HIV, malaria and anemia, for which school-age children are not a sentinel population. When testing for HIV, DHS and AIS surveys generally sample women of reproductive age (15–49 years) and males aged 15–59 years. For anemia, women of reproductive age and children aged 6–59 months are sampled, while for malaria only children aged 6–59 months are included. For STH and schistosomiasis, the highest burden of infection is among school-age children; as a result, survey coverage of this age group would have to be expanded if surveys such as the DHS were to become viable platforms for post-elimination surveillance. Sentinel populations for post-elimination surveillance of other NTDs must be confirmed to ensure that survey sample collection includes appropriate age groups.

### Biomarkers

At present, surveillance for NTDs is dependent on a disparate range of biomarkers, most not well suited for post-elimination surveillance. For example, in areas endemic for *Wuchereria bancrofti* filariasis, WHO recommends the Alere Filariasis Test Strip (FTS), which measures circulating filarial antigen in human blood. However, the FTS is cross-reactive with Loa loa antigens [27], which limits its utility for post-elimination surveillance in (previously) coendemic settings. STH and schistosomiasis are routinely detected using microscopy-based diagnostic tools that count the number of parasite eggs excreted in urine or stool. However, the low sensitivity of these methods when infection intensities are very low means they are considered inadequate for elimination surveillance [28].

Diagnostics, however, are likely to keep improving as programs progress, and TPPs that specify the minimum and ideal characteristics for diagnostics for post-elimination surveillance are already published for schistosomiasis [29], STH, and trachoma and are in development for lymphatic filariasis [25, 29]. According to published TPPs, acceptable post-elimination surveillance diagnostics for schistosomiasis, STH, and trachoma would be based on detection of antibodies in finger-stick blood samples that could be used with minimal or no infrastructure [25].

**Figure 3.**
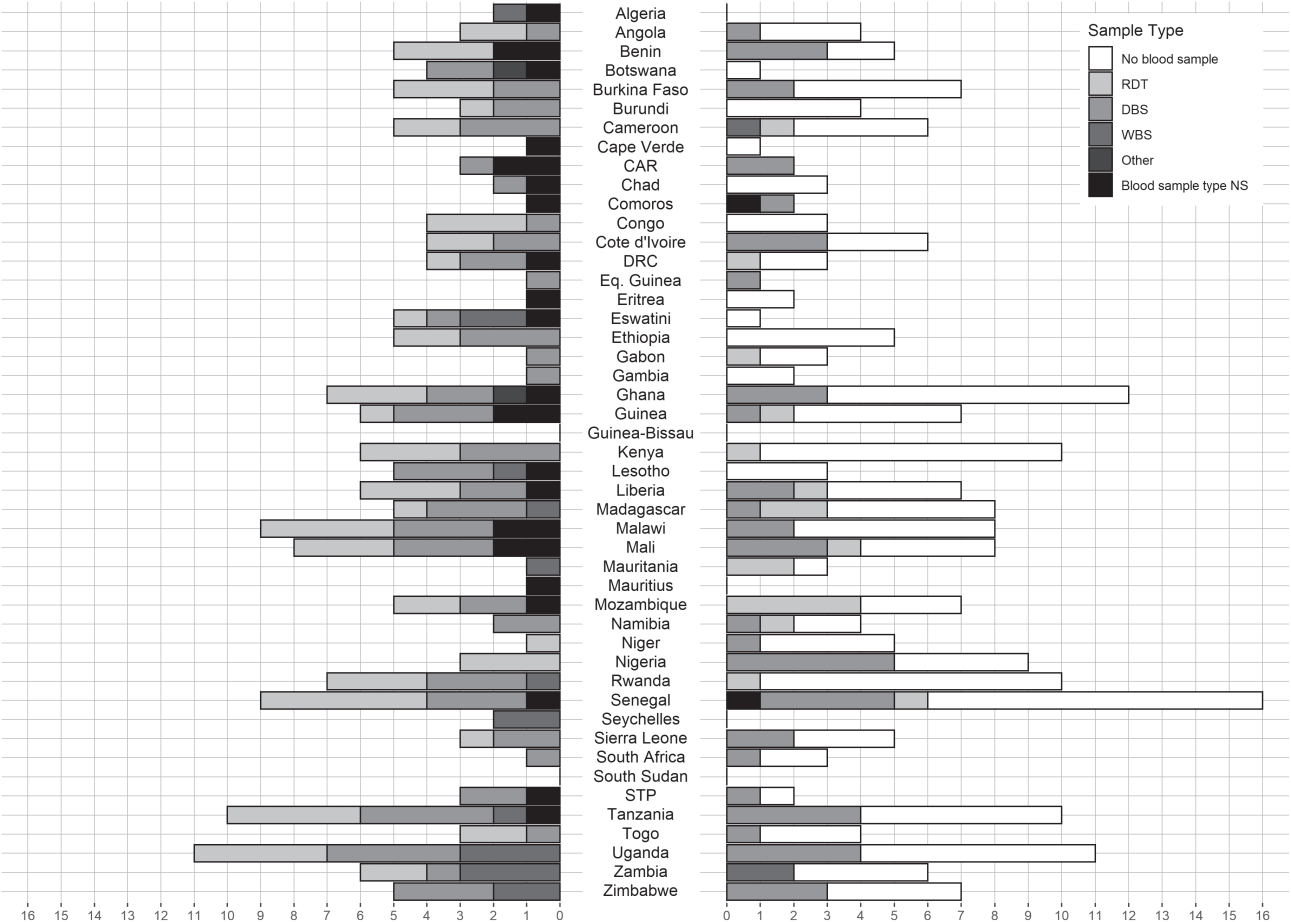
Number of large-scale surveys ever conducted by country for the WHO Africa region. Left side shows the number of all surveys that collected blood samples by sample type. Right side shows all demographic and health surveys ever conducted, whether they collected blood samples and sample type, where relevant. Where multiple samples were taken, surveys using WBS were categorized as WBS, those using a mixture of DBS and RDT were categorized as DBS, and those using RDT and other types of tests were categorized as RDT. Abbreviations: CAR, Central African Republic; DBS, dry blood spot; DRC, Democratic Republic of Congo; NS, not stated; RDT, rapid diagnostic test; STP, Sao Tome and Principe; WBS, whole blood sample.

Considering a scenario where future diagnostics measure blood or sera-based biomarkers for all NTDs included within the surveillance platform, it should be noted that dried blood spots (DBS) were collected by 101 surveys (55.0%) and whole blood by just 18 (10.8%) of identified blood surveys (Figure 3). The relatively high coverage of DBS is encouraging, as this would facilitate the application of seroepidemiological methods that have been previously applied to NTDs including trachoma, lymphatic filariasis, schistosomiasis, and onchocerciasis in research settings [26, 30–33]. The development and validation of multiplex serological assays further increase the operational feasibility of serological techniques in surveillance by enabling measurement of a broad range of responses with high repeatability from limited blood samples [34].

## WAYS FORWARD AND CONCLUSIONS

Crucially, ongoing post-elimination NTD surveillance must be at a low cost to maintain government and donor commitment to diseases that will no longer be regarded as public health issues. NTD surveillance integrated within existing systems will maintain low costs of monitoring and strengthen current infrastructure, using existing passive or active surveillance systems, or a combination of the 2. Lessons can be learned from other disease areas. Countries that have successfully eliminated malaria, for example, have typically relied on a combination of both passive and active case detection, with staff dedicated to surveillance and integrated response mechanisms [11], coupled with integration within DHS platforms.

Inclusion in DHS or future alternative representative surveys will require adjustments to both the DHS and the NTD programs. In order to better understand these adjustments and provide a clearer characterization of optimal post-elimination surveillance, we offer some considerations for future work. First, an increase in the number of epidemiological studies at fine spatial scale conducted within countries that previously had NTDs as a public health problem and in areas very close to elimination thresholds is needed. These studies would provide greater insight into the extent and importance of spatial heterogeneity at low prevalence settings. Second, increasing the number of samples collected from individuals outside the usual age groups (eg, during impact assessment surveys) would help to ensure that age infection profiles are better defined and that appropriate sentinel populations identified. Third, to test mathematical transmission models of recrudescence and to understand the population-level seroreversion rate needed for serological surveillance approaches, longitudinal or repeat cross-sectional studies conducted several years apart are needed.

Existing large-scale survey platforms offer enormous potential for integrated surveillance, and so it is perhaps unsurprising that they are already overloaded with questions. Given the increasing potential of integrated serosurveillance, it is likely that in time blood spots will likewise become overloaded with requests. To provide sufficient justification for inclusion of NTDs, it will be essential that we improve our operational research evidence base, including target product profiles for new diagnostics, to take full advantage of these possibilities.

## Supplementary Data

Supplementary materials are available at *Clinical Infectious Diseases* online. Consisting of data provided by the authors to benefit the reader, the posted materials are not copyedited and are the sole responsibility of the authors, so questions or comments should be addressed to the corresponding author.

## Notes

***Acknowledgments.*** We thank Graham Medley for helpful discussions.

***Supplement sponsorship.*** This article appears as part of the supplement “Sustainable control of neglected tropical diseases – surveillance and diagnostics”, sponsored by the NTD Modelling Consortium.

***Financial support.*** This work was supported by the Bill and Melinda Gates Foundation through the Neglected Tropical Disease Modelling Consortium (grant OPP1184344).

***Potential conflicts of interest.*** All authors: No reported conflicts of interest. All authors have submitted the ICMJE Form for Disclosure of Potential Conflicts of Interest. Conflicts that the editors consider relevant to the content of the manuscript have been disclosed.
